# LGI1-antibody encephalitis: how to approach this highly treatable dementia mimic in memory and mental health services

**DOI:** 10.1192/bjp.2024.72

**Published:** 2024-05-03

**Authors:** Sophie N. M. Binks, Adam Al-Diwani, Adam E. Handel, Tomasz Bajorek, Sanjay Manohar, Masud Husain, Sarosh R. Irani, Ivan Koychev

**Affiliations:** Oxford Autoimmune Neurology Group, Nuffield Department of Clinical Neurosciences, University of Oxford, UK; and Department of Neurology, John Radcliffe Hospital, Oxford University Hospital NHS Foundation Trust, Oxford, UK; University Department of Psychiatry, University of Oxford, UK; Department of Psychological Medicine, John Radcliffe Hospital, Oxford University Hospital NHS Foundation Trust, Oxford, UK; and Oxford Community Psychological Medicine Service, Oxford Health NHS Foundation Trust, Oxford, UK; Oxford Autoimmune Neurology Group, Nuffield Department of Clinical Neurosciences, University of Oxford, UK; Department of Neurology, John Radcliffe Hospital, Oxford University Hospital NHS Foundation Trust, Oxford, UK; Department of Psychological Medicine, John Radcliffe Hospital, Oxford University Hospital NHS Foundation Trust, Oxford, UK; and Oxford Community Psychological Medicine Service, Oxford Health NHS Foundation Trust, Oxford, UK; Department of Psychological Medicine, John Radcliffe Hospital, Oxford University Hospital NHS Foundation Trust, Oxford, UK; Department of Neurology, John Radcliffe Hospital, Oxford University Hospital NHS Foundation Trust, Oxford, UK; Department of Experimental Psychology, University of Oxford, UK; and Nuffield Department of Clinical Neurosciences, University of Oxford, UK; Department of Neurology, John Radcliffe Hospital, Oxford University Hospital NHS Foundation Trust, Oxford, UK; Department of Experimental Psychology, University of Oxford, UK; and Nuffield Department of Clinical Neurosciences, University of Oxford, UK; Oxford Autoimmune Neurology Group, Nuffield Department of Clinical Neurosciences, University of Oxford, UK; and Department of Neurology, John Radcliffe Hospital, Oxford University Hospital NHS Foundation Trust, Oxford, UK; University Department of Psychiatry, University of Oxford, UK; and Department of Psychological Medicine, John Radcliffe Hospital, Oxford University Hospital NHS Foundation Trust, Oxford, UK

**Keywords:** Dementia, neuroimmunology, leucine-rich glioma-inactivated 1, psychosis, older adults

## Abstract

Leucine-rich glioma-inactivated 1-antibody-encephalitis is a treatable and potentially reversible cause of cognitive and psychiatric presentations, and may mimic cognitive decline, rapidly progressive dementia and complex psychosis in older patients. This aetiology is of immediate relevance given the alternative treatment pathway required, compared with other conditions presenting with cognitive deficits.

There has been growing recognition of antibody-mediated causes of disturbance to brain and behaviour – the ‘autoimmune encephalitides’.^1^ The underlying pathophysiology is driven by autoantibodies to surface epitopes of key central nervous system proteins.^1^ They disrupt synaptic and circuit function, which left untreated can progress to sustained tissue damage and cognitive and neuropsychiatric disability. Although not common, most autoimmune encephalitis subtypes are highly immunotherapy-responsive, creating urgency for clinical recognition and instigation of disease-modifying treatments.^2^

Within psychiatric practice, N-Methyl-D-aspartic acid receptor-antibody encephalitis (NMDAR-Ab-E) has garnered most attention since it can take the form of a severe mental illness in its earliest stages.^3,4^ A lesser-known autoimmune encephalitis with particular relevance to older adult psychiatry is leucine-rich glioma-inactivated 1-antibody encephalitis (LGI1-Ab-E).^5^ It has a comparable incidence to NMDAR-Ab-E and is the most common autoimmune encephalitis above the age of 40.^1,6^ Its median onset is in people in their mid-60s, and usually presents with amnesia, personality change and focal seizures.^1,5^ Like NMDAR-Ab-E, it is highly modifiable by immune-targeted treatments, but untreated can leave a deficit comparable to dementia.^7^ Typically memory deficits are prominent,^8,9^ with a characteristic pattern of mixed anterograde and retrograde memory loss.^10^ This may be accompanied by executive function impairment, as well as wide-ranging neuropsychiatric impacts, including anxiety, depression and emotional lability (pathological tearfulness).^1,8,9,11^ Sleep disturbance may occur, including insomnia and a dream enactment/rapid eye movement (REM) disorder.^8^ LGI1-Ab-E cohort studies provide evidence supporting early immunotherapy in abrogating seizures and reducing the risk of developing a fixed cognitive deficit.^7^

In LGI1-Ab-E, two-thirds of patients are male, and tumours are rare.^7^ A hallmark of the condition is faciobrachial dystonic seizures (FBDS). These involve a brief jerk of the hemi-face and/or ipsilateral upper limb and/or lower limb. They are present in two-thirds of cases and are pathognomic if observed.^1,7^ However, FBDS and other focal seizures can be missed because of brevity. FBDS can also be mistaken for myoclonus and therefore interpreted as suggesting a diagnosis of Creutzfeldt–Jakob Disease (CJD).^12,13^ Depression/anxiety, paranoia, hallucinations and anxiety occur in at least 50% of people with LGI1-Ab-E^8^ and, conversely, temporal lobe seizures can mimic panic or anxiety attacks.^1^

This analysis will focus on LGI1-Ab-E presentations which may result in referral to older adult mental health teams. Routine antibody testing without clinical suspicion is not recommended. Rather, this clinical approach should help prompt antibody testing where the pre-test probability is sufficiently raised.

## Case 1: cognitive decline

A 76-year-old man, a semi-retired postal worker, had an onset of personality change and cognitive decline over several months. He had become less argumentative, a departure from his formerly strong-minded and reportedly bullish character. This was accompanied by various cognitive deficits including episodic memory (forgetting that he had taken a train journey), topographical memory (getting lost in familiar places) and anterograde memory (losing track and being repetitive in conversation). His family also described him as having ‘vague and distant’ spells up to four times a day, during which he would be less responsive but not lose consciousness, followed by momentary disorientation. Three months after symptom onset, he was admitted initially under acute medicine with delirium and hyponatraemia (126 mmol/L). Because of historic dependent alcohol use, an initial working diagnosis of Alzheimer’s disease and concurrent Wernicke’s encephalopathy was made, and he was treated with intravenous (IV) thiamine.

Following assessment by cognitive neurology, a collateral history emerged that he had been abstinent for 20 years. On cognitive testing, there was evidence of executive dysfunction (unable to perform the Luria 3-stage sequence and concrete interpretation of proverbs), no ideomotor apraxia or apraxia for meaningless gestures, and normal speech and verbal working memory. He was able to reason about similarities. Addenbrooke’s Cognitive Examination III (ACE-III) was 83/100, with some points lost on memory (20/26), including one point on encoding. However, visuospatial function was normal with no marks lost on cube, infinities and clock components. LGI1-Ab-E was therefore deemed by the cognitive neurology consultant to be a more probable unifying diagnosis than Alzheimer’s disease, given preserved parietal function but evidence of episodic memory deficit, perhaps suggestive of selective hippocampal dysfunction or dorsolateral prefrontal cortex impairment. He had a lumbar puncture which showed a normal cell count and mildly elevated protein (599 mg/L). Magnetic resonance imaging (MRI) depicted mesial temporal lobe T2/FLAIR hyperintensity with slight left-sided swelling. These changes were consistent with LGI1-Ab-E. LGI1-autoantibodies were detected in both serum and cerebrospinal fluid (CSF). After treatment with high dose steroids and plasma exchange (PLEX), he improved. Following a brief period of neurorehabilitation, he returned to employment and independent living. At last review, his cognition had improved (ACE-III 93/100).

## Case 1 discussion

In this case, autoimmune encephalitis clues included hyponatraemia – present in 60–70% of LGI1-Ab-E due to autoantibody-binding to LGI1-expressing, anti-diuretic hormone (ADH)-secreting neurons^7^ – and subtle focal seizures.^12^ Seizures are observed in up to 20% of people with Alzheimer’s disease, but usually as a late feature.^14^ As the work-up proceeded, more phenotypic features favouring autoimmune encephalitis emerged, including sub-acute (days to weeks) onset and the pattern of neuro-cognitive and -behavioural deficits. This degree of neurocognitive dysfunction coming on over a few months, together with a lack of constructional and ideomotor apraxia and the presence of intact visuospatial skills lowered the likelihood of Alzheimer’s disease, a simple and useful clinical test.^15^

A recent study from a tertiary cognitive clinic using stringent criteria identified that 0.8% of people could be diagnosed with exclusive or concurrent autoimmune encephalitis (Table 1).^16^ All were stated to have clinical features inconsistent with dementia, such as subacute or fluctuating disease course. However, the same researchers have highlighted that up to 50% of patients >45 years old with LGI1-Ab-E could still fulfil dementia diagnostic criteria. These patients did not have prominent seizures, MRI or CSF abnormalities, and 71% presented with cognitive decline.^12^

Unlike NMDAR-Ab-E, in which definitive diagnosis relies upon the detection of NMDAR-autoantibodies in CSF,^2^ LGI1-antibodies are not always found in CSF with usual testing methods.^8^ The rate of serum LGI1-antibodies of uncertain clinical significance in healthy and disease control populations is low (<0.1%).^17^ Serum LGI1-antibody testing should be considered with an atypical case such as this one.

## Case 2: rapidly progressive dementia mimicking CJD

A 90-year-old male retired scientist deteriorated over a few months. He developed a prominent movement disorder, frequent falls, cognitive decline and incontinence. He could no longer drive or live independently, and required a Zimmer frame and daily help. The illness began with abnormal jerking movements of his right side, spreading to bilateral involvement, and he then developed orofacial dyskinesias. There were no hallucinations. His personality changed, becoming prone to aggressive outbursts.

On examination, he was disinhibited and had word-finding difficulties, answering questions with repetitive and circumlocutory speech, and full neurological examination was impossible. A rapidly progressive dementia (RPD) was considered, including sporadic CJD. Dementia screening bloods were unremarkable. CT head was within normal limits for age, and MRI brain showed severe bilateral hippocampal atrophy, with no medial temporal lobe high signal and was reported as consistent with Alzheimer’s disease. LGI1-antibodies were strongly positive in serum but negative in the CSF. Montreal Cognitive Assessment (MoCA) improved from 22/30 (with points lost in trail making, indicative of executive dysfunction, attention, and all five marks for delayed recall) to 30/30 with immunotherapy. However, ongoing psychiatric input, including aripiprazole, was needed for residual persistent behavioural challenges. Continued challenging behaviour led to a residential care placement.

## Case 2 discussion

Autoimmune encephalitis is an important differential diagnosis of RPD, a small (3–4%) and clinically challenging type of dementia.^13^ As noted above, FBDS (often described by individuals as jerks, jolts or spasms) may be confused with the myoclonus of CJD, most typical in the sporadic form. One study found that ∼20% of people with LGI1-Ab-E and FBDS were suspected of having CJD during their disease course.^18^ Features favouring FBDS over myoclonus include stereotypy, and recurrence up to hundreds of times a day;^1^ also in LGI1-Ab-E, as this case illustrates, FBDS frequently precede cognitive impairment.^7^ This is unlikely with myoclonus and CJD, where it is a late feature.

An autoimmune cause may underlie up to 13% of non-CJD RPD seen in specialist clinics.^19^ This and other early studies examined antibodies directed against the voltage-gated potassium channel (VGKC) complex. This test has since been superseded by more specific cell-based assays for LGI1, the predominant antigenic target in central nervous system presentations, and also contactin-associated protein 2 (CASPR2) antibodies.^5,20^ These antibodies can also cause encephalitis but commonly drive peripheral nerve disease. A variety of surface autoantibodies, including to LGI1, have been found in up to ∼6% of post-mortem RPD patients, further accentuating the importance of excluding a reversible pathology in these people.^21^

Brain atrophy was found in both people with LGI1-Ab-E initially thought to have dementia in Bastiaansen et al.^16^ The patient described in case 2 had bilateral hippocampal atrophy which could be consistent with Alzheimer’s disease or with limbic encephalitis (LE) in its later stages. However, despite its possibility, the question of concurrent Alzheimer’s disease was not explored further, so it is possible he had both conditions. Although he did improve from a cognitive perspective, which would be less consistent with Alzheimer’s disease, he remained neurobehaviourally disturbed and did not return to independent living.

In the Oxford Autoimmune Neurology Service, out of 60 LGI1-Ab-E patients, nearly 20% had moderate to moderately severe disability and loss of functional independence. Also, few working-aged individuals (4/27, 15%) were able to return to their previous employment.^9^ Thus the outcomes in LGI1-Ab-E, while generally good, remain not ‘good enough’ with both delayed recognition and treatment, and unmet need for more effective therapeutics, likely to contribute.^22^

## Case 3: cognitive decline and psychosis

A 67-year-old retired male gardener, living alone with no prior psychiatric or forensic history, was witnessed by friends to have an aggressive verbal outburst. Over the next 4 months, this was followed by becoming quieter, withdrawn and forgetful. He developed frequent short-lived moments of reduced responsiveness lasting 3–5 s. He was also reported to have filled his house with cardboard boxes, was restless, spent excessively, and repetitively counted paper clips (most likely punding – a problem with impulse control in keeping with the overall neurobehavioural presentation). He had less need for sleep, his self-care deteriorated and he became unconcerned about not eating. Acquaintances observed episodes of left-hand jerking movements and facial contortion lasting up to 10 s. During these, there was clouding of consciousness. His GP organised routine blood tests, including electrolytes, and MRI brain, which were unremarkable. The community psychiatry team diagnosed mania and prescribed olanzapine, but he was non-compliant with treatment. He then began conversing with unseen individuals he believed to be living in his house, reporting people were holding parties with cats coming out of the walls and believed that pictures were talking to him. He continued to be disinhibited including leaving the house dressed as a pirate or even naked, and he was found trying to stop traffic. This led to a compulsory in-patient psychiatric admission 14 months after the start of symptoms.

On the ward, he continued to have the same left-sided jerks every few hours as well as frequent falls. Based on the presence of visual hallucinations and falls, dementia with Lewy bodies (DLB) was considered. However, symptomatic treatments were of little help. Around 3 months into the admission, the prominent ongoing left-sided jerks prompted the in-patient team to test serum for LGI1-antibodies. After a positive result, and liaison with the medical team, he was admitted to the local general hospital for high dose IV steroids. A repeat MRI brain now demonstrated bilateral T2 high signal in the medial temporal lobes, consistent with LE. Routine CSF examination, not including neurodegeneration markers, was unremarkable. After 5 days of IV steroids, there was notable improvement. On day 6, his friends were able to hold a normal conversation with him, and he had no further seizures.

Six months later, during oral steroids taper, overspending and impulsive decision-making returned. He improved with an increase of steroids alongside aripiprazole. Four years post-onset, his ACE score is 94/100, and all anti-seizure and anti-psychotic medications have been successfully withdrawn.

## Case 3 discussion

This case presented with many LGI1-Ab-E hallmark features: a man in his mid-60s with a prominent neurobehavioural syndrome encompassing relatively rapid onset of personality and behavioural change, cognitive decline and seizures. However, the presence and relevance of temporal lobe seizures (brief moments of behavioural arrest) and FBDS (left hand jerks and facial contortion lasting seconds) was initially overshadowed by the prominent behavioural changes and major risks to himself and others. Subtle seizures are overlooked in a quarter of people, as reported in in a cohort of 48 patients with LGI1-Ab-E.^12^ Frequent falls are another clue.^1^ A further flag to an autoimmune case is the fast progression; only CJD approaches the tempo seen in patients with autoimmune encephalitis.^13^ Psychotic symptoms occur in about a fifth of people with LGI1-Ab-E.^8^ Psychotic features are well known in DLB, but here the speed of onset, lack of fluctuations and absence of Parkinsonian features pointed away from this.^13^ Lack of response to antipsychotic medication has also been suggested as a trigger to consider an autoimmune cause.^23^ In contrast to NMDAR-Ab-E, there is no reported increased sensitivity or antipsychotic malignant-like syndrome in LGI1-Ab-E. Initial MRI brain imaging was normal, but this may be the case in up to 30–40% LGI1-Ab-E, especially if performed early.^8^

This individual’s illness was responsive to first-line immunotherapy, in LGI1-Ab-E high dose steroids often with PLEX or IV immunoglobulins. Observational evidence shows adding immunotherapy to anti-seizure medications brings about rapid seizure cessation and halts progression to cognitive decline.^7^ Rituximab, a CD20 monoclonal antibody which depletes B cells, is gaining traction for LGI1-Ab-E relapse,^24^ having observational support for this in NMDAR-Ab-E.^25^

Four years post-onset, his ACE score is 94/100, and all anti-seizure and anti-psychotic medications have been successfully withdrawn, but he reports ongoing significant and functionally limiting fatigue. In a published cohort of 60 LGI1-Ab-E patients, fatigue was the most common chronic problem in >50%,^9^ and the most important determinant of quality of life.^26^ Alongside this, widespread neuropsychiatric difficulties were found. This included cognitive difficulties in a third of patients surveyed, as well as increased anxiety in a third and depressive symptoms in a fifth.^9^ These findings highlight the need for multidisciplinary rehabilitation beyond the acute illness phase, with psychiatric alongside neurology input together with other therapies.

## Conclusion

LGI1-Ab-E is a treatable and potentially reversible cause of cognitive and psychiatric presentations that is immediately relevant to older adult psychiatric practice. A unifying feature of our cases is of a prominent neurobehavioural syndrome, with disinhibition and apathy of various forms, alongside global neurocognitive deficits. The tempo of LGI1-Ab-E means that it is an important differential diagnosis of RPDs such as CJD that are unmodifiable. Failure to consider it in a timely fashion could therefore have a significant impact on prognosis. Sometimes-overlooked clues can be subtle non-motor focal seizures or FBDS, often manifesting prior to cognitive impairment, or episodes that may appear syncopal, and hyponatraemia. Memory is the most frequent cognitive domain impacted, with a characteristic mixed anterograde and retrograde pattern. In addition, there may be behavioural disturbance. MRI brain may be unrevealing, particularly in the earliest stages of disease. However, many patients show signal change in the medial temporal lobes and atrophy of this region with progression. A serum antibody test is sufficient as an initial investigation in most patients (Box 1). Suspected or confirmed cases are managed under neurology with further investigations including CSF analysis and electroencephalogram (EEG), but psychiatry retains an ongoing role as part of the multidisciplinary team (Box 1).

## Continuing professional development – suggested further reading

A new research paper using stringent criteria to identify patients with autoimmune encephalitis from a tertiary memory clinic can be found in a work by Bastiaansen et al. (2023).^16^

A case-based focus on causes of rapidly progressive dementia, including LGI1-Ab-E can be found in a work by Day (2022).^13^

Practical tips on readily executable bedside assessment of parietal lobe function, including of apraxia can be found in a work by Tabi & Husain (2023).^15^

A recent review of a broad range of autoimmune encephalitides in a clinically-oriented journal can be found in a work by Uy, Binks and Irani (2021).^1^

## Supplementary Material

Supplementary material is available online at https://doi.org/10.1192/bjp.2024.72

Supplementary materials

## Figures and Tables

**Table 1:** Autoantibodies either detected as dementia mimics in a Dutch memory clinic cohort, or which can present with psychiatric-predominant syndromes. Reproduced with adaptations from Table 1 of Uy et al ^a^

A: Entities represented in the Dutch cohort^16^ (7/920 patients in total, 0.8%)						
Antigenic target	Number (%) in Dutch cohort	Median onset age, years (range)	Sex ratio (M:F)	Core clinical features	Psychiatric notes	MRI findings
DPPX	1 (0.1%)	53 (13–76)	1.5:1	Multifocal encephalitis with myoclonus, tremors and exaggerated startle response, with prominent diarrhoea / weight loss	May have fluctuating course. Mental state manifestations include amnesia, delirium, psychosis and depression	Normal or non-specific
IgLON5	3 (0.3%)	64 (46–83)	1:1	4 main syndromes: sleep disorder (REM and NREM parasomnias, sleep apnoea); bulbar syndrome; PSP-like syndrome; cognitive syndrome ± chorea	May be a chronic disease course (months to >12 years described). Movement and sleep disorder may be more prominent than cognitive changes and lead to consideration of PSP	∼80% normal/non-specific; ∼15% brainstem atrophy; 5% bilateral hippocampal atrophy
LGI1^8^	2 (0.2%)	64 (31–84)	2:1	LE with frequent focal seizures, including characteristic FBDS	Amnestic syndrome with mixed anterograde and retrograde (esp. biographical, adjacent to/inclusive of illness) memory loss. Cognitive decline (∼70%), personality change (∼35%), hallucinations (∼20%) and affective symptoms (up to 30%)	∼75% abnormal. ∼40% increased signal/swelling in medial temporal lobes
NMDAR^4,12^	1 (0.1%)	21 (2 months–85 years)	1:4	Encephalitis with polysymptomatic neuropsychiatric presentation, polymorphic movement disorder, language disorder, autonomic dysfunction, coma, central apnoea	Usual onset with severe psychiatric disorder in young adults, with ovarian teratoma in ∼30% of women <45; less commonly as a ‘dementia mimic’	70–80% normal or non-specific, with a typical LE in a minority
B: Selected other entities with potential to present to psychiatric services						
Antigenic target	Proportion with dementia-like presentations^b^	Median onset age, years (range)	Sex ratio (M:F)	Core clinical features	Psychiatric notes	MRI findings
AMPAR	Not established	Mean 53.1 (14–92)	2:1	LE with prominent confusion, amnesia, seizures and psychiatric/behavioural symptoms	Acute/sub-acute onset, including acute-onset psychosis; psychiatric symptoms may be associated with diagnostic delay. Malignancy (thymoma/lung) in around 2/3s	∼85% abnormal (67% with bilateral mesial temporal FLAIR hyperintensities)
CASPR2	∼15% >45 years old may fulfil dementia criteria	66 (25–77)	9:1	LE and Morvan’s syndrome (triad of peripheral nerve hyperexcitability, insomnia and autonomic instability, often with psychiatric features)	Typically sub-acute onset with mental state changes including cognitive decline (∼80%), amnesia (∼70%), behavioural disturbance (∼60%) and psychosis (∼33%)	∼30% increased signal in medial temporal lobes
GABAaR	Not established	40 (2 months–88 years)	1:1	Encephalitis with frequent status epilepticus	Characteristic for potential to affect all age groups and distinctive MRI features; seizures almost unanimously present, often with cognitive (∼70%) and behavioural (∼50%) disturbance	>80% cortical and subcortical FLAIR signal abnormalities involving 2+ brain regions
GABAbR	∼15% >45 years old may fulfil dementia criteria	61 (16–77)	1.5:1	LE with prominent seizures. 50% with tumour, mainly small cell lung	May present as a rapidly progressive dementia (∼15%); almost all with behavioural/cognitive disturbance and 1/3 with hallucinations	∼70% abnormal (45% increased signal in medial temporal lobes)

AMPAR, α-amino-3-hydroxy-5-methyl-4-isoxazolepropionic acid receptor; CASPR2, contactin-associated protein-like 2; DPPX, dipeptidyl peptidase-like protein 6; F, female; FBDS, faciobrachial dystonic seizures; FLAIR, fluid-attenuated inverted recovery; GABA_A/B_R, Gamma aminobutyric acid A/B receptors; IgLON5, immunoglobulin-like cell-adhesion molecule 5; LE, limbic encephalitis; LGI1, Leucine-rich glioma-inactivated 1; M, male; MRI, magnetic resonance imaging; NMDAR, N-Methyl-D-aspartic acid receptor; PSP, progressive supranuclear palsy; REM/NREM, rapid eye movement/non-rapid eye movement.

a This is an open access article distributed under the terms of the Creative Commons CC BY 4.0 licence, which permits unrestricted use, distribution and reproduction in any medium, provided the original work is properly cited. Additional references used in this version and not in the main reference list are included in supplementary information.

b Data from retrospective Dutch cohort of Bastiaansen et al (2021).^12^ Table replicated with additions from Uy et al (2021).^1^

## Data Availability

Data availability is not applicable to this article as no new data were created or analysed in this study.

## Box 1 Investigations and shared management practice in LGI1-Ab-E

**Serological testing**

For serum autoantibody testing, a standard serum tube is required and can be sent as routine. We recommend requesting LGI1-antibodies; VGKC antibodies are no longer in clinical use.^20^ Turnaround is usually within a few days to a few weeks, and cost in the UK is in the region of £50. Some mental health service blood-ordering systems may not offer LGI1-antibodies as a routine option. Discussion with local clinical laboratory sciences providers can facilitate adding this to the available list of choices.

**Management**

A referral to the local specialist neurology service in suspected cases is the suggested route in high suspicion cases (e.g. positive antibodies, history of frequent seizures or very rapid functional decline). The subsequent management is best achieved through neurology-psychiatry multidisciplinary teams. An example of this in our region is a regular multidisciplinary team meeting with neurology, psychiatry and laboratory medicine experts that reviews suspected cases referred to psychiatric services and makes recommendations about management. In addition, a liaison neurologist does consults within psychiatry services ∼ one day per week, seeing patients with a broad range of potential neurological presentations, including when autoimmune encephalitis is a differential diagnosis. This commissioned service includes performing lumbar punctures if clinically necessary.
