# Supplementary material for: LGI1-antibody encephalitis: how to approach this highly treatable dementia mimic in memory and mental health services
Source: Br J Psychiatry. Author manuscript; Available in PMC 2024 Jun 1. (PMC7615977; doi:10.1192/bjp.2024.72)
Supplement: Supplementary materials [file EMS194724-supplement-Supplementary_materials.docx]

**Supplementary materials – additional references for Table 1, by antigenic target**

**DPPX**

Tobin WO, Lennon VA, Komorowski L, Probst C, Clardy SL, Aksamit AJ, et al. DPPX

potassium channel antibody: Frequency, clinical accompaniments, and outcomes in 20

patients. Neurology. 2014;83(20):1797–803.

**IgLON5**

Sabater L, Gaig C, Gelpi E, Bataller L, Lewerenz J, Torres-Vega E, et al. A novel non-rapid-

eye movement and rapid-eye-movement parasomnia with sleep breathing disorder

associated with antibodies to IgLON5: A case series, characterisation of the antigen, and

post-mortem study. Lancet Neurol. 2014;13(6):575–86.

**LGI1**

Binks SNM, Klein CJ, Waters P, Pittock SJ, Irani SR. LGI1, CASPR2 and related antibodies:

a molecular evolution of the phenotypes. J Neurol Neurosurg Psychiatry. 2018;89(5):526-34.

**AMPAR**

Laurido-Soto O, Brier MR, Simon LE, McCullough A, Bucelli RC, Day GS. Patient

characteristics and outcome associations in AMPA receptor encephalitis. J Neurol.

2019;266(2):450–60.

Graus F, Boronat A, Xifro X, Boix M, Svigelj V, Garcia A, et al. The expanding clinical profile

of anti-AMPA receptor encephalitis. Neurology. 2010 Mar 9;74(10):857–9.

**CASPR2**

Van Sonderen A, Ariño H, Petit-Pedrol M, Leypoldt F, Körtvélyessy P, Wandinger KP, et al.

The clinical spectrum of Caspr2 antibody-associated disease. Neurology. 2016;87(5):521–8.

**GABA_A_**

Spatola M, Petit-Pedrol M, Simabukuro MM, Armangue T, Castro FJ, Barcelo Artigues MI, et

al. Investigations in GABA_A_ receptor antibody-associated encephalitis. Neurology. 2017 Mar

14;88(11):1012–20.

**GABA_B_**

Van Coevorden-Hameete MH, De Bruijn MAAM, De Graaff E, Bastiaansen DAEM, Schreurs

MWJ, Demmers JAA, et al. The expanded clinical spectrum of anti-GABABR encephalitis

and added value of KCTD16 autoantibodies. Brain. 2019;142(6):1631–43.
